# Percutaneous Endoscopic Gastrostomy Tube Insertion in Patient With Situs Inversus Totalis

**DOI:** 10.7759/cureus.14650

**Published:** 2021-04-23

**Authors:** Abdulwahab Hamid, Muneera Almohannadi, Ahmad Badi, Betsy Varughese, Saad Rashid Mohammad Al Kaabi

**Affiliations:** 1 Gastroenterology and Hepatology, Hamad Medical Corporation, Doha, QAT; 2 General Internal Medicine, Hamad Medical Corporation, Doha, QAT; 3 Gastroenterology, Hamad Medical Corporation, Doha, QAT

**Keywords:** gastrostomy, situs, inversus, totalis, endoscopic

## Abstract

Situs inversus totalis (SIT) is a rare congenital condition in which the abdominal and thoracic organs are completely reversed from right to left, and their diagnosis is usually incidental. However, patients with SIT need a comprehensive radiological evaluation before undertaking any invasive procedures. Percutaneous endoscopic gastrostomy (PEG) insertion is an effective procedure for enteral feeding in patients with difficulty swallowing. Many post-procedural complications have been reported after the PEG procedure.

We performed PEG insertion in an 85-year-old Qatari SIT patient, who was admitted to the hospital as a case of aspiration pneumonia and on nasogastric feeding tube (NGT). The procedure was started while the patient was in left lateral decubitus position as in normal anatomy patients after careful examination and in accordance with the general principles of PEG insertion. No complications were seen, neither intraoperative nor postoperative in two months follow-up.

We suggest that in a patient with SIT, PEG insertion can be performed while the patient is in left decubitus position with no additional risk or extra intraoperative time if the pre-operative anatomical position of vital organs is carefully evaluated.

## Introduction

Situs inversus (SI) is an abbreviation of “situs inversus viscerum,” which is a Latin phrase that stands for a rare disease that leads to a mirror image organization of the organs in the abdomen and chest [1-3]. It is called situs inversus totalis (SIT) when there is a complete transposition (right to left reversal) of the thoracic and abdominal organs, which constitute a mirror image of the normal anatomy [2,4]. The heart is not present in the left chest in its usual position, but it is on the right side called dextrocardia [5]. The exact cause of SI has not been understood [6,7].

The prevalence of SI varies among various populations. However, it has been estimated to affect less than one in 10,000 [8]. Usually, there are no specific symptoms related to SI, as the only difference is a change in the position of organs without a change in the functional status. Mostly, a random x-ray taken for an unrelated cause leads to an incidental finding of this abnormality. Its clinical significance relates to the fact that due to the transposition of the inner organs, symptoms, and signs elicited during physical examination are present on the atypical side of the body in such patients. SI is usually an autosomal recessive genetic disorder. However, it can be linked to X-chromosomes. It may also be found in identical twins [9]. Complete situs inversus or SIT has been found to appear once in approximately 6,000-8,000 births [10].

Percutaneous endoscopic gastrostomy (PEG) is the best choice for medium- and long-term enteral feeding for patients who have difficulty in swallowing. PEG was first introduced in 1980 when endoscopy was used to insert a feeding tube into the stomach [11]. Usually, access to the gastrostomy tube can be achieved using endoscopy, radiological imaging, or surgical techniques. Because of low cost, less invasiveness, and less general anesthesia requirement, which is a challenging variable in vulnerable patients in whom gastrostomy tubes are most frequently inserted, PEG is considered a better option than surgical methods to insert a feeding tube [12,13].

## Case presentation

**Figure 1:**
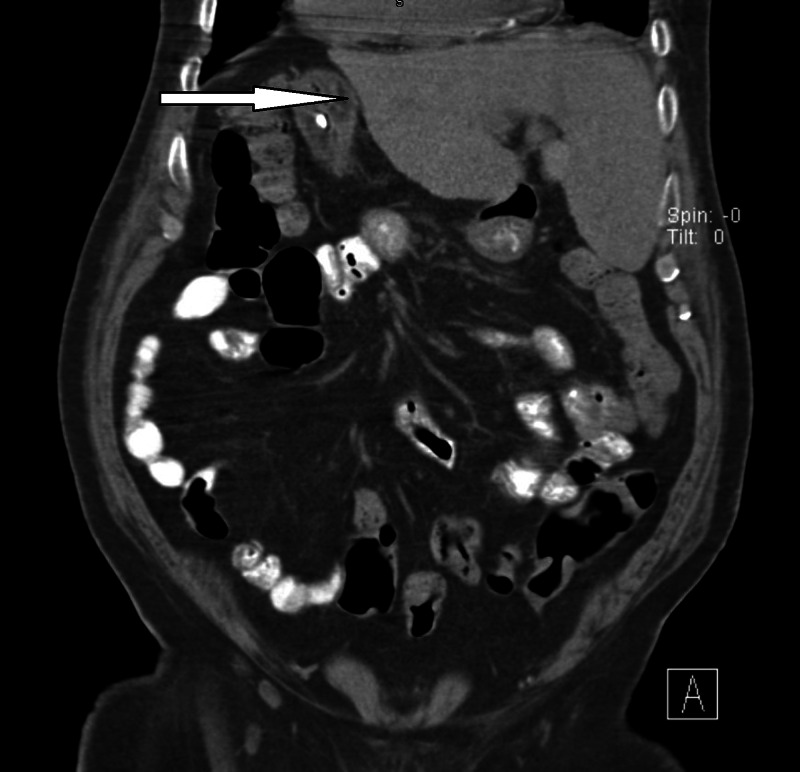
Coronal CT scan abdomen of a patient with SIT Arrow pointed to the liver, located on the left side in a patient with SIT. SIT, Situs inversus totalis.

An 85-year-old Qatari male was admitted to the hospital as a case of aspiration pneumonia and managed accordingly. He is bed-bound and unable to swallow for few years. He was on NGT feeding. The family discussed the issue of difficulty in managing the NGT and requested PEG tube placement as many times the patient was intolerant of NGT and tried removing it. The details regarding the possible complications of PEG were discussed, and the family was made aware that it will not prevent aspiration pneumonia. The family consent was obtained after stabilization of the patient and achievement of his basic health status. A chest x-ray and abdominal ultrasound were taken during his admission, which diagnosed complete SIT. A CT scan of the chest and abdomen was further performed (Figure 1) to have a full picture of the anatomy of the internal organs, which confirmed the diagnosis of SIT. 

**Figure 2:**
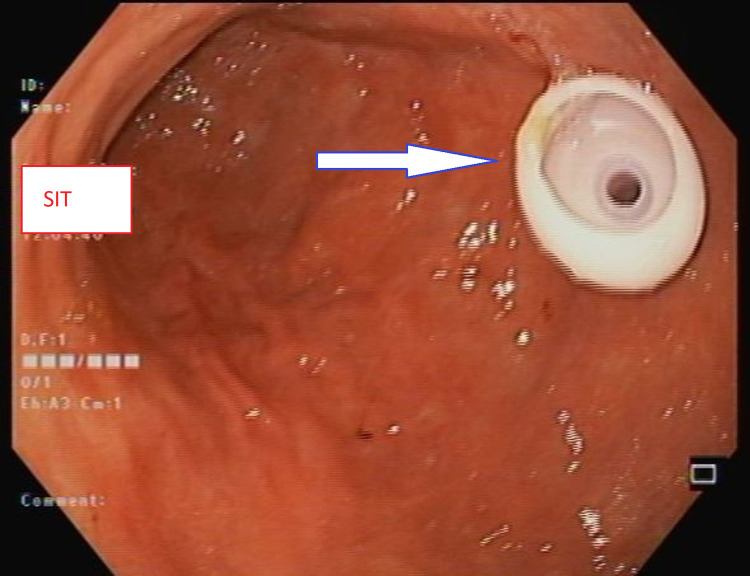
The inner part of PEG fixed in the stomach Arrow pointed to the internal part of PEG. PEG, Percutaneous endoscopic gastrostomy.

One hour prior to the procedure, the patient had received one gram of IV ceftriaxone. On examination, he was clinically and biochemically fit for the procedure. The patient was connected to a cardiac monitor and placed in the left lateral decubitus position. The mouthpiece was inserted in place. Then the procedure was started under conscious sedation. The scope passed down through the esophagus to the stomach. There were no mucosal abnormalities identified, and the scope passed easily to the duodenal bulb. Then the scope turned counterclockwise to pass to the second part of the duodenum, which showed normal mucosa, and after that, the scope was withdrawn back to the stomach, and the trans-illumination was identified through the anterior abdominal wall, few centimeters below the xiphoid process and to the right of the midline. A puncture site was chosen with the help of an endoscopic light after manually pressing the abdominal wall to find the thinnest site. Subsequently, a gastrostomy feeding tube (PEG-24-Pull; Wilson-Cook Medical GI Endoscopy, Winston-Salem, NC, USA) was inserted into the anterior wall of the lower gastric body (Figure 2). Hemostasis was secured, the air was sucked out, scope withdrawal was performed without any complications, and the feeding through PEG started on the next day.

## Discussion

Insertion of PEG is considered as an effective procedure for patients with difficulty in swallowing, where a feeding tube is inserted through the anterior abdominal wall into the stomach with the assist of endoscopy [11]. Post-procedural complications continue to be reported, including surgical site infection followed by gastric bleeding, pneumoperitoneum, feeding tube displacement, aspiration pneumonia, intestinal perforation, peritonitis, necrotizing fasciitis, buried bumper syndrome, etc. [14]. However, in our patient, there were no complications noted, neither intraoperative nor postoperative in two months follow-up.

While performing PEG in patients with SIT, the procedure needs more caution because of the abnormal anatomical placement and the organs as well as because of postoperative complications. Endoscopic retrograde cholangiopancreatography (ERCP) and endoscopy are usually performed in patients with SIT in the right lateral decubitus position, considering the reversed abdominal organs [15]. In contrast, in this patient, we performed the procedure when the patient is in the left lateral decubitus position, and there was no difficulty encountered during the procedure. The previous study reported that when endoscopy and colonoscopy were performed in a patient with SIT in the left lateral decubitus position as in ordinary patients with careful planning and in compliance with the general principles, the procedures were performed successfully without pain or complications. Though there were no procedural complications, the procedure took longer time in patients with SIT as compared to non-SIT patients [16]. We did not find such observation during our procedure as the time taken for the procedure was almost the same as that of the patient without SIT.

## Conclusions

Esophagogastroduodenoscopy and PEG insertion can be carried out in a patient with SIT while the patient is in left lateral decubitus position with no additional risk or extra intraoperative time if the preoperative anatomical position of vital organs is carefully evaluated.
